# MATSAP: An automated analysis of stretch-attend posture in rodent behavioral experiments

**DOI:** 10.1038/srep31286

**Published:** 2016-08-09

**Authors:** Kevin S. Holly, Casey O. Orndorff, Teresa A. Murray

**Affiliations:** 1 Department of Biomedical Engineering, Louisiana Tech University, Ruston, Louisiana, United States of America; 2 Department of Mathematics and Statistics, Louisiana Tech University, Ruston, Louisiana, United States of America

## Abstract

Stretch-attend posture (SAP) occurs during risk assessment and is prevalent in common rodent behavioral tests. Measuring this behavior can enhance behavioral tests. For example, stretch-attend posture is a more sensitive measure of the effects of anxiolytics than traditional spatiotemporal indices. However, quantifying stretch-attend posture using human observers is time consuming, somewhat subjective, and prone to errors. We have developed MATLAB-based software, MATSAP, which is a quick, consistent, and open source program that provides objective automated analysis of stretch-attend posture in rodent behavioral experiments. Unlike human observers, MATSAP is not susceptible to fatigue or subjectivity. We assessed MATSAP performance with videos of male Swiss mice moving in an open field box and in an elevated plus maze. MATSAP reliably detected stretch-attend posture on par with human observers. This freely-available program can be broadly used by biologists and psychologists to accelerate neurological, pharmacological, and behavioral studies.

Rodent behavioral analysis is often used to assess the effects of pharmaceuticals, implanted devices, or surgical procedures in preclinical research. The development of image analysis tools has enabled researchers to quantitatively assess various rodent behaviors quickly and objectively^1^,^2^. However, most automated scoring programs track patterns in spatial locomotor exploration and neglect ethological behaviors, such as head dipping and stretch-attend posture (SAP)^1^. Currently, there is no accurate tracking and scoring software that can directly detect SAP^3^.

SAP, which is generally associated with anxiety, occurs when the rodent lowers its back, elongates its body, and is either standing still or moving forward very slowly^4^. SAP is a naturally occurring behavior found in rodents, such as mice, etc. that can be reliably intensified by certain experimental paradigms, such as placing the rodent in an open-field test^4^,^5^. In mice, the SAP behavior occurs when the mouse is undergoing risk-assessment specifically due to an internal exploratory-anxiety conflict. It can also occur under fearful risk-assessment where SAP would be an ambivalent element reflecting an approach-avoidance tendency^4^,^6^. When SAP is present during a passive avoidance situation, mice approach or avoid the object at nearly equal rates, which indicates they are undergoing risk-assessment during an approach-avoidance conflict^5^,^6^. SAP is a good identifier for conflict behavior in mice and can be used to evaluate the effects of drugs at reducing these internal conflicts^7^. During exploratory-anxiety conflict situations, SAP can be used as a valid measure of anxiety as anxiolytic drugs have successfully reduced SAP^5^,^7^,^8^,^9^.

SAP has been evaluated in elevated plus maze (EPM)^10^, open field (OF)^11^, rat exposure test^12^, and canopy stretch attend posture test^8^. Increased SAP behavior of rodents near the entrance of the open arms in EPM and along the border of the canopy in the canopy stretch attend posture test has demonstrated SAP as a risk assessment behavior^8^,^13^.

In classical anxiety tests, such as EPM and OF, the conventional spatiotemporal measurements may not detect effects of novel anxiolytic medications^10^. SAP has been found to be more sensitive to the effects of classical and atypical anxiolytics than traditional spatiotemporal indices in the murine plus-maze^14^,^15^. For example, SAP is especially sensitive to the effects of ligands acting on 5-HT1A receptors^14^,^15^. It is hypothesized that SAP can be related more to the cognitively oriented aspects of anxiety^14^. Inclusion of ethological measurements such as SAP in EPM provides a more comprehensive profile on the anxiolytic or anxiogenic effects of a treatment^9^,^15^,^16^. SAP can also help differentiate between anxiogenesis and sedation effects of drugs^1^,^17^. Despite finding that risk assessment measurements are more sensitive to anxiety modulating drugs than traditional indices, Carobrez *et al*. found that only a quarter of studies have adopted them^1^.

SAP is usually evaluated using its frequency of occurrence^8^,^18^,^19^, although some have quantified SAP in both duration and frequency^11^,^13^,^15^. Researchers to date have scored ethological behaviors manually either with the aid of Observer XT^20^,^21^,^22^ or without computer-aided assistance^16^,^23^,^24^,^25^. Some investigators are even using EthoVision XT for spatiotemporal measurements while using trained observers for manually recording ethological behaviors such as SAP^26^,^27^,^28^,^29^. Evaluation with human observers is time-consuming and susceptible to error as people become fatigued and lose concentration during long mental tasks. Human observers may introduce subjectivity into their scoring leading to variable interpretations of observed behaviors between individual observers which decreases inter-observer reliability^30^. Intra-rater variability is also a concern because human observers may have different scores for the same videos when blindly scoring the same set of videos twice. In contrast, computers are consistent and measure objectively. Further, computers do not experience fatigue or require training.

Event-recording programs such as Hindsight and EthoVision require a user to manually press a button when the behavior of interest occurs. These programs facilitate the viewing and counting of the behavior; this process is time consuming and subject to human error. While commercially available software could be used to automatically detect SAP with additional customization, this can be costly for the purchaser. Currently, EthoVision XT can be used to find the speed and elongation of a rodent to detect SAP if the user is given the proper threshold values and if the software is modified to provide the additional output. However, to the best of our knowledge, the use of this program to detect SAP has not appeared in the literature. To encourage a greater use of SAP as a metric in behavioral tests, we have created an open source MATLAB-based software, MATSAP, to detect SAP.

## Results

**Figure 1:**
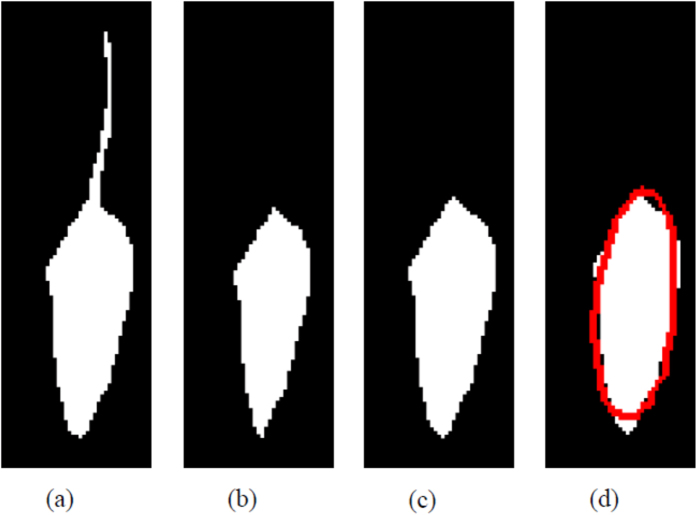
The MATSAP software opens a multi-TIFF video of the mouse and then (**a**) makes a binary image of the rodent, (**b**) erodes the image which eliminates the tail, (**c**) creates a dilated image that bring size of the rodent back to normal, and then (**d**) places an ellipse around the body of the rodent.

MATSAP detects SAP by generating an ellipse fitted around a rodent in multi-TIFF video files and then it uses the calculated eccentricity value of the ellipse along with the rodent’s calculated speed to discriminate SAP from running. MATSAP provides results in Excel and American Standard Code for Information Interchange (ASCII) files for importing into statistical programs. It also displays plots of the eccentricity, speed, and SAP detection over time. The program also provides optional features, which include a threshold preview screen to aid the user in selecting the appropriate threshold values to convert the multi-TIFF images into binary images for analysis, visualization of the image analysis (Fig. 1), and saving the visualized output (Supplementary Video S1). If desired, these features can be used for sample videos in a large batch and then the batch can be run without them or with periodic sampling to reduce run times. Thus, MATSAP is flexible to allow for the optimization of runtimes.

### Open field performance

To test the ability of MATSAP to detect SAP, ten 5-minute videos at 10 frames per second (fps) of white mice moving in an OF box with a dark background were evaluated. Five blinded human observers (inter-rater reliability 0.83) established a “ground truth” consensus score of SAP in the videos on a second by second basis. SAP results from MATSAP were compared to this human consensus. A minimal frame rate of 10 fps is common for detecting spatiotemporal transitions; thus, this rate was used to test MATSAP’s functionality^31^. An inter-rater reliability above 0.80 is considered an excellent agreement beyond chance according to Fleiss *et al*. and in almost perfect agreement according to Koch and Landis^32^,^33^. Based on the human consensus score, SAP was present in 337 seconds (*Positive*) and was not present in 2663 seconds (*Negative*). The accuracy, sensitivity, and specificity of MATSAP when compared to the human consensus score can be found in Table 1. The average accuracy of the individual observers was 92.7 ± 3.4% (mean ± SD) compared to the consensus score. The F-score was 66.7% and the Matthews correlation coefficient (MCC) was 0.64. MCC is preferable over the F-score because the *Positive* and *Negative* classes are imbalanced^34^. Since the MCC is closer to 1 than −1, a strong positive relationship is indicated between MATSAP’s classification of SAP and the classification by the human consensus^34^,^35^. The chosen eccentricity and speed thresholds of 0.90 and 12 cm/s, respectively, were verified by Receiver Operating Characteristic (ROC) curves (Supplementary Fig. S11 and S12). The area under the ROC curve (AUC) was 0.8802 (Supplementary Table S1), which is rated “very good”^34^.

SAP duration and frequency as calculated by MATSAP was compared to the human consensus for each of the 10 OF videos. The duration for the human consensus was 33.7 ± 6.9 s (mean ± SD), while MATSAP was 31.1 ± 8.9 s (mean ± SD). No significant difference was found between MATSAP and the human consensus; t(9) = −1.02, p = 0.33. SAP frequency for the human consensus was 1.1 ± 0.4 per min (mean ± SD), while MATSAP was 2.9 ± 1.9 per min (mean ± SD). There was a significant difference in the SAP frequency results between MATSAP and the human consensus; t(9) = 3.18, p = 0.01.

### Elevated plus maze performance

We employed a similar procedure for an EPM experiment. Nine 10-minute videos at 10 fps of male Swiss mice in a maze with a dark background were evaluated by 5 blinded human observers (inter-rater reliability 0.86) to reach a “ground truth” consensus score. Based on the human consensus score, SAP was present in 2059 seconds (*Positive*) and was not present in 3341 seconds (*Negative*). The accuracy, sensitivity, and specificity of MATSAP in detecting SAP in EPM compared to the human consensus can be found in Table 1. The average accuracy of the individual observers was 87.4 ± 6.4 (mean ± SD). The F-score was 80.97% and MCC was 0.69. Again, MCC indicated a strong positive relationship between MATSAP’s classification of SAP to the classification by the human consensus. The chosen eccentricity and speed thresholds of 0.89 and 8 cm/s were verified by ROC curves (Supplementary Figs S13 and S14). The AUC was 0.8470 (Supplementary Table S2), which is within the “very good” range^34^.

The speed threshold was lower than the threshold for the OF test as the mice had less space to gain momentum with only 5-cm wide paths. The eccentricity value was lower because the mice would exhibit SAP while bending out toward the open arms. This posture generates a shorter ellipse during SAP. An eccentricity value of 0.90 would fail to detect some of these bent SAP postures, so an eccentricity of 0.89 was used to increase the sensitivity although this change decreased specificity.

SAP duration and frequency as calculated by MATSAP was compared to the human consensus for each of the 9 EPM videos. The duration for the human consensus was 225.8 ± 33.4 s (mean ± SD), while MATSAP was 166.3 ± 41.3 s (mean ± SD), with a significant difference; t(8) = −4.15, p = 0.003. The frequency results for the human consensus was 3.7 ± 0.4 per min (mean ± SD), while MATSAP was 5.2 ± 0.6 per min (mean ± SD), which was significantly different; t(8) = 6.53, p < 0.001.

### Runtimes

The runtime for MATSAP to analyze the videos, as measured by the in-build MATLAB profiler, was markedly shorter than the evaluation time used by the human observers. On a laptop with an Intel^®^ Core™ i7-3520M core processer at 2.90 GHz and with 6.00 GB of RAM, the runtime for a 5-min video at 10 fps was less than 2 min when the output was visualized at 1 fps without saving the video. Furthermore, the runtime was only about 30 s without the visualized output or saving videos. These runtimes exclude the time spent by the user to answer prompts and to use the threshold preview screen. If the parameters (video dimensions, fps, and threshold) are the same, the user can set the program to run continuously through hundreds of videos. Depending on the human observer’s skill level, the evaluation time ranged from 10 to 45 min per 5-min video.

## Discussion

Currently, there is no commercially available behavioral analysis software that directly detects SAP or any free software that can be readily utilized to detect SAP. Due to limited funding in some research labs, there is a need for inexpensive software that detects SAP in rodents. To meet this need, a freely available, open source software program with a flexible, user-friendly GUI called MATSAP was successfully developed to detect SAP. The program runs in a basic MATLAB installation with the Image Processing Toolbox™. MATSAP allows users to analyze multi-page Tag Image File Format (multi-TIFF,.tif) video files of rodents from an overhead view. MATSAP was a reliable program for detecting SAP when using male Swiss mice weighing 32.5–40.0 g in both the OF and EPM.

Computers are quick, consistent, and tireless, unlike human observers. The flexibility of the program and the user-friendly interface allows for optimal run times. It takes less than 2 minutes to analyze a 5-minute, 10-fps video using MATSAP. In contrast, it would take a human observer from 10 to 45 minutes depending on the observer’s skill level. This 5- to 23-fold decrease in time means that the program is well suited for online applications. Furthermore, MATSAP can replace human observers with the exception of an occasional check of the program’s output.

Mean frequency values were higher when using MATSAP versus the human consensus. It is possible that MATSAP could be more accurate than the human consensus, which was assumed to be the “ground truth”. The observers may be influenced by the psychological effect known as the law of closure^44^. If there was a small break between SAP behaviors, the human observers may have considered the separate events as one. MATSAP would discriminate these events as separate resulting in a higher frequency, but maintaining roughly the same duration of the behavior as observed in the case of the OF test. This suggests that MATSAP would provide a more sensitive frequency measurement.

**Figure 2:**
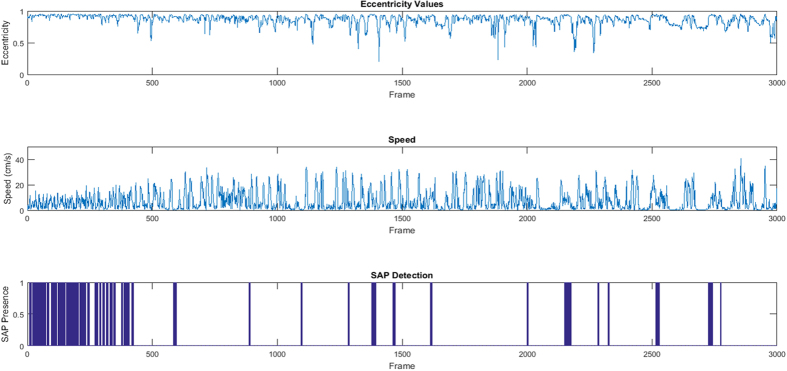
MATSAP output plots for an open field test. The eccentricity values (top panel), speed (middle panel), and the detection of SAP (dark blue areas, bottom panel) are shown for each frame in the video.

MATSAP detected a lower duration of SAP than the human consensus in EPM. It is possible that MATSAP was not detecting SAP behavior while the mice were bending around a corner peering into an open field arm. The generated ellipse may not have been long enough to pick up all of these instances. The frequency of SAP was slightly higher in EPM than the OF as indicated by both MATSAP and the human consensus results. There were more transitions between non-SAP and SAP behaviors throughout the EPM test in comparison to the OF test (Fig. 2, Supplementary Fig. S1). This could have increased the likelihood of discrepancies among the human observers leading to the lower average accuracy of the individual observers.

### Flexibility of software

MATSAP is flexible to meet different research requirements. For example, speed and eccentricity threshold parameters can be adjusted to achieve greater sensitivity or specificity, as needed. As long as there is a visual contrast between the rodent and the background, MATSAP can detect SAP. This includes a white rodent on black surfaces, a dark rodent on white surfaces, and an infrared video of a rodent on a cool surface. Furthermore, the program can run on different computer operating systems provided that a MATLAB release is available for the operating system.

Another aim of this program was to have a graphical user interface that makes the program easy to use, especially for optimizing the program runtime for scoring videos. To do this, MATSAP begins with a series of questions that guide the user in selecting the level of visualization that occurs when the program is running. This visualization allows the operator to verify that the program is running properly. To reduce the runtime for all videos, the operator has the option of displaying only 1 frame of the video per second or not at all. Saving the video output is also optional. Additionally, MATSAP is designed to display the fewest dialog boxes possible between videos. The output files are saved in both Excel and ASCII. If the Excel files fail to save, MATLAB has a built-in function to automatically save them as a Comma Separated Values (CSV,.csv) file. If all of the parameters (thresholds, dimensions, and fps) are the same for a set of videos in a computer file folder, the user can apply them to all videos within that folder, so that the program becomes fully automated without any additional input. This allows the user to analyze hundreds of videos without further input.

### Uses and limitations

MATSAP may also be useful for other behavioral tests such as the canopy test and the rat exposure test. With a slight modification, MATSAP could be used to quantify forward SAP (F-SAP). This would be useful in tracking SAP towards an unfamiliar object, such as in novel object recognition tests^36^, or away from a novel object, such as an electrifiable prod 5. Combining SAP detection with spatiotemporal measures could help differentiate between ‘protected’ (when the rodent is under a covering or in an enclosed area) and ‘unprotected’ SAP 8,11,12,15,18,19.

The eccentricity threshold value of 0.90 can only be confidently used for Swiss male mice with a weight range of 32.5–40.0 g. Different strains of mice or rats may have different threshold values for speed and eccentricity. Differences in sex, species, strains, weight and genetic modifications would require the use of different eccentricity and speed threshold values^3^. These can be calculated using the Threshold Optimizer. Another limitation is that the current version of MATSAP (v1.0) only works in offline mode. This is because video files require preparation, such as cropping videos to a known scale and converting videos to multi-TIFF files before running MATSAP.

### Future work

Our goal is to create a collaborative user group whose participants will provide their optimized eccentricity and speed threshold parameters for other rodents, strains of mice, and possibly larger animals. These would be curated and posted on a user forum. Tables of these threshold values would also contain the species, age, weight, sex and genetic mutations associated with the parameter values. Once these tables are established, MATSAP can be modified to include a user-input option to select the type of animal, strain, weight, and genetic modification in order to automatically select the appropriate threshold values. Additionally, we plan to develop real-time analysis capabilities so that users can obtain results while performing an experiment.

As mentioned in the Uses and Limitations section, MATSAP may also be useful for other behavioral tests and these should be evaluated. Additionally, we plan to develop online, real-time analysis capabilities where the user can obtain instant results while performing an experiment by using a camera with firewire that can connect directly to a computer running MATSAP. User region selection will be implemented so the user can select a region of known dimensions or a region to crop. This feature can also reduce video preparation time if offline analysis is desired. Saving output videos files without using the MATLAB getframe function is preferred, so the user can save a video of the visualized output without having to display it (thus decreasing the runtime). Another future improvement will be to allow the program to read other video formats so the user does not need to convert files into multi-TIFFs.

## Conclusion

MATSAP provides a quick, easy, and reliable method to detect the more sensitive rodent anxiety measure, SAP, in less time and with less potential subjectivity than the human observers. The program offers a user-friendly graphical interface and a flexible structure that caters to individual needs and that facilitates the optimization of runtimes. MATSAP enables scoring a large quantity of rodent behavioral videos in a relatively short period of time. This is an advantage when testing a large number of rodents. Future work is needed to establish eccentricity and speed thresholds tables for different rodent species, strains and sizes, as well as to evaluate other ethological behaviors. This can be accomplished by curating software users’ parameters and additional open-source companion programs akin to the user-provided plug-ins for the free image processing software, ImageJ^37^.

## Methods

### Animals

Ten male Swiss mice from Jackson Laboratories were used in this study housed in groups of 4–6 mice per cage to avoid stress and anxiety induced by social isolation^38^, although some believe individual housing decreases anxiety in mice^13^. The mice were housed in a 12-hour day/night cycle where food was administered *ad libitum*. Behavioral test procedures were approved by the Louisiana Tech University Institutional Animal Care and Use Committee and were in accordance with the National Institute of Health Guide for the Care and Use of Laboratory Animals (NIH Publications No. 80–23, revised 1996).

### Behavioral experiments

Four month old male Swiss mice were used in this study (n = 10 for OF, n = 9 for EPM). The weight of the male mice ranged from 32.5–40.0 g during testing. Behavioral tests were conducted by a male experimenter. Five observers evaluated the recorded videos of the experiment. For each mouse, there was a minimum of 24 hours between each behavioral test.

### Open field test

The OF test apparatus consisted of an open square wooden container (30 × 30 cm) with 25-cm high walls enclosing the perimeter. The walls and floor of the container were spray painted black. On the floor of the container, a white 16 square grid was drawn^39^. A camera mounted above the box and facing perpendicular to the floor was used to record the movement of the mice at 29 fps in high-definition MPEG Transport Stream (MTS,.mts) video format. To begin the test, a mouse was placed in the corner of the container. The mouse was allowed to explore the container for 5 minutes before being removed and placed into its home cage. Between trials, super hypochlorous water was used to clean the floor and walls of the container^3^.

### Elevated plus maze

An EPM was built from medium density fiber board using previously established dimensions^2^. Two opposing open arms and two opposing enclosed arms extended 25 cm from a 5 × 5 cm central platform forming a plus shape. Enclosed walls were 25 cm tall and the maze was elevated 50 cm above the floor. To begin the test, a mouse was placed in the central platform facing the south enclosed arm and was allowed to move freely about the maze for 10 minutes. This time period was chosen as opposed to 5 minutes to allow ample time for the mice to explore the open arms^2^. A ceiling-mounted video camera facing perpendicular to the floor with a field of view centered on the central platform recorded mouse movement at 29 fps in MTS video format. The platform of the maze was black to provide color contrast to the white Swiss mice and the testing room was illuminated with standard fluorescent lights. Between trials, the central platform and all four arms were cleaned with super hypochlorous water to remove odors left by the previous mouse^2^.

### Video preparation

Videos were converted from MTS files at 29 fps to Audio Video Interleave (AVI,.avi) files at 10 fps without audio and then loaded into ImageJ 1.47t, a public domain image processing program^37^. Videos were converted to grayscale and cropped to the dimensions of the apparatus (i.e. OF or EPM) with a 1:1 width to height ratio based on pixel count. Before saving as a multi-TIFF file, an image of the apparatus without a mouse present was added as the last frame of the video to use for background subtraction in the other frames so that the white rodent is readily distinguished from a black background.

### MATSAP availability

The most recent version of MATSAP can be found at [https://www.mathworks.com/matlabcentral/fileexchange/58412-matsap]. MATLAB (Version R2012a or greater) along with the Image Processing Toolbox™ is required to run MATSAP. Comments are embedded within the MATLAB code that explain steps, such as analyzing images and optimization steps.

### Structural design of software

The software allows the users to analyze multi-TIFF video files of rodents from an overhead view. The user will need to convert video files to multi-TIFF files with the last frame being the background. Conversion to TIFF files can be done through the public domain image processing program ImageJ if the video format is AVI^37^.

**Figure 3:**
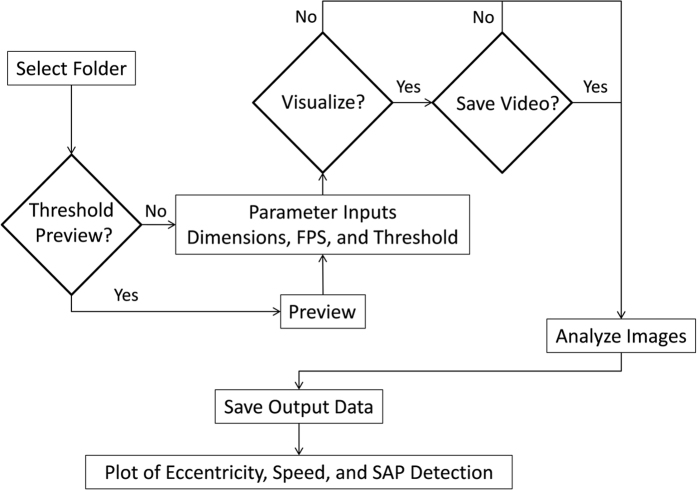
Simplified flowchart of MATSAP program that shows the optional features and work flow.

**Figure 4:**
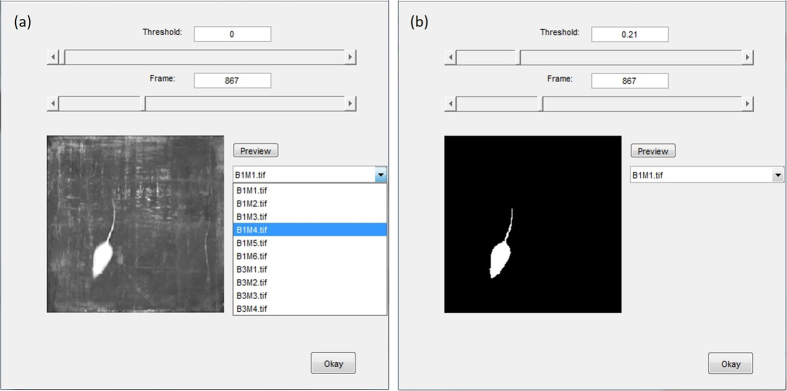
The MATSAP Threshold Previewer allows the user to select the appropriate threshold value to create a high contrast binary image. (**a**) TIFF image from original video. (**b**) Same image frame after threshold has been set to create a binary image.

Figure 3 is a simplified flowchart of the software program. When the user runs MATSAP, a dialog box will prompt the user to select the folder containing the multi-TIFF files for analysis. The user will be asked if the rodents are darker than the background in case the images need to be inverted for analysis. Then the program will give the user the option of opening the MATSAP Threshold Previewer, an interactive preview screen of the videos, to test for an appropriate binary conversion threshold value that will be used to convert the images into a highly contrast binary images (Fig. 4). After viewing the optional threshold preview or selecting “No” in the dialog box, another dialog box appears for the user to input parameters of the video, which includes dimensions of the area covered in the video, the fps, the binary conversion threshold value, and the speed and eccentricity threshold values. After this, the user is given the options “yes” or “no” to have a visualized output of the analysis (Supplementary Fig. S2). If “yes” is selected, then the option of saving the video is presented to the user. The images are then analyzed and the user is prompted to select the file folder location to save an Excel file containing SAP detection results. Plots of the eccentricity, speed, and SAP detection are displayed and saved in the directory folder (the folder that contained multi-TIFF files for analysis) unless an alternative directory is chosen for output files. The data are also archived in ASCII files in case a user does not have Excel in the default directory, unless the user specifies otherwise. A result summary spreadsheet is also generated containing the total SAP percentage, time, and frequency for each video in the directory folder. If all the input parameters are the same, including the binary conversion threshold value, the dimension, and the fps, the user has the option of skipping subsequent input dialog box prompts for the remainder of the videos in the folder. Furthermore, the user can set MATSAP to run until all of the videos in the folder are analyzed.

### Image analysis

After the user enters the required parameters and answers the dialog prompts, MATSAP begins its fully automated image analysis by subtracting the background from each image frame and then converting the frames into black and white, binary images (Fig. 1a). The rodent’s tail is removed by eroding the perimeter of the rodent with the built-in imerode MATLAB function. This step creates an ellipse-like form, but it also reduces the overall size of the mouse (Fig. 1b). In earlier versions of this program, the eccentricity values were distorted by the length and changing position of the tail. This step removes the tail and results in more accurate eccentricity values. In some frames, a small stub of the tail is visible. This did not adversely affect detection of SAP. After this automated step, the program restores the binary image of the rodent to its normal size by dilating the perimeter of the rodent with the imdilate function, thus restoring the original girth and length, but without the tail (Fig. 1c)^40^. MATSAP then selects the largest object with the aid of the regionprops MATLAB function and generates an ellipse around it as described in Steve Eddin’s 2010 MathWorks blog post, “Steve on Image Processing.” This is done by first finding the longest length of the object and denoting it as the major axis. A perpendicular vector is then created from the centroid of the object and denoted as the minor axis length. With these two lines, the ellipse is generated around the body of the mouse (Fig. 1d). MATSAP provides an option to display the generated ellipse on the image frames so that the user can verify that the program is working properly.

The elongation of the rodent is denoted by the eccentricity value of the generated ellipse. Based on these eccentricity values, a SAP detection array is formed on a frame by frame basis with “1” being the value when SAP is present and “0” when SAP is not present. If the ellipse’s eccentricity is greater than 0.90 (or another user-specified eccentricity threshold) at an individual time-step, then the SAP detection array is given the value of “1” at the current time-step.

The speed of the rodent is found by using the centroid values of each frame provided by the regionprops command. The pixel distance between the centroids in consecutive frames is calculated. Based on the video’s field of view dimensions provided by the user, the actual distance between the centroids is found. The speed of the rodent is then determined using this actual distance multiplying this by the video’s fps rate. If the rodent’s speed is greater than 12 cm/s (or another specified speed threshold) at an individual time-step, then the SAP detection array is given the value of “0” at the current time-step. This eliminates the cases where the mouse is not in a SAP but is elongated because it is running. The assumption is made that SAP cannot occur in a time duration less than or equal to 0.5 seconds. So to eliminate false positives, any time there are consecutive 1’s of a length less than or equal to half of the fps in the SAP detection array, these ones are changed to zeroes.

The program provides plots displaying SAP detection as well as the corresponding speed and eccentricity values (Fig. 2). The plots are saved and the data are archived in Excel and ASCII files. A summary result spreadsheet is also generated providing the duration, percentage, and frequency of SAP for each the multi-TIFF file. If selected, videos of the image analysis are saved as well.

### Evaluation methods

Ten 5-min videos at 10 fps of male Swiss mice in an OF box maze and nine 10-min videos at 10 fps of male Swiss mice in an EPM were evaluated by 5 human observers that were blinded to each other and MATSAP. Both the inside of the OF box and the EPM were painted black for maximizing contrast with the white mouse. For each second of the videos, the 5 observers determined if SAP was present giving a score of “1” if present and “0” if not. A human consensus score was determined via majority voting of each of the individual observers at each second. The SAP detection software developed scores of the videos frame by frame. After running MATSAP on MATLAB (Version R2012a), the results were translated from frame-based scoring to a time-based scoring (in s). SAP was considered present in a specific second if SAP was detected in at least one frame. MATSAP has a built-in filter for removing the durations of SAP less than a half second to minimize false positives. The second-based SAP detection array of 1’s and 0’s generated by MATSAP was compared to the human consensus SAP detection array; the human consensus was treated as the ground truth.

To determine the runtime of MATSAP, MATLAB profiler was utilized. A typical laptop was used with an Intel^®^ Core™ i7-3520M core processer at 2.90 GHz and with 6.00 GB of RAM. The runtime of MATSAP for a single video was measured at two different settings, obtaining results (i) with the visualized output being displayed and without saving the video and (ii) without displaying the output visual or saving the video.

### Statistical analysis

Using R software along with the inter-rater reliability package (‘irr’ Package), a two-way agreement, average-measure, intra-class correlation was used to compute the inter-rater reliability of the 5 human observers that established the ground truth^41^. The accuracy, sensitivity, and specificity of MATSAP compared to the human consensus (ground truth) were determined along with the F-score, MCC, and area under the curve (AUC). Binomial proportion confidence intervals for the accuracy, sensitivity, and specificity were calculated using normal approximation interval (Wald interval) since the sample size (the total seconds of video evaluated) was greater than 30 and the proportions were not close to 0 or 1^42^. The AUC was approximated by the simple trapezoidal method; see equation (1)^34^,^43^.


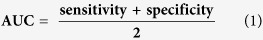


The MCC plots and ROC curves were generated with a custom script written in MATLAB to justify the selection of speed and eccentricity thresholds used to conclude if SAP was present in an image frame. In the ROC curves, which were also generated in Excel, the most optimal threshold would be located in the top left of the graph (Supplementary Fig. S11–S14) as this is where sensitivity and specificity are the highest. For the MCC plots, the speed and eccentricity threshold values that provided the maximum MCC would be the most optimal, albeit also containing a reasonable sensitivity and specificity ratio. A paired-samples t-test was conducted to compare each video scored by MATSAP to the consensus of 5 observers for that video. This was performed for both SAP frequency and duration measurements.

## Additional Information

**How to cite this article**: Holly, K. S. *et al*. MATSAP: An automated analysis of stretch-attend posture in rodent behavioral experiments. *Sci. Rep*. **6**, 31286; doi: 10.1038/srep31286 (2016).

## Supplementary Material

Supplementary Information

Supplementary Video

## Figures and Tables

**Table 1:** Performance measurements for MATSAP compared to human consensus.

Apparatus	Accuracy (%)	c.i. (%)	Sensitivity (%)	c.i. (%)	Specificity (%)	c.i. (%)
OF	90.5	89.1–91.9	84.9	79.9–89.9	91.2	89.8–92.6
EPM	85.6	84.4–86.8	86.0	82.8–89.2	85.3	84.0–86.6

Abbreviation: confidence interval, c.i.
