# Supplementary material for: MATSAP: An automated analysis of stretch-attend posture in rodent behavioral experiments
Source: Sci Rep. 2016 Aug 9;6:31286. doi: 10.1038/srep31286 (PMC4977506; doi:10.1038/srep31286)
Supplement: Supplementary Information [file srep31286-s1.pdf]

MATSAP: An Automated Analysis of Stretch-attend Posture in Rodent Behavioral Experiments

Kevin S. Holly[1], Casey O. Orndorff[2], and Teresa A. Murray[1*]

1. Department of Biomedical Engineering, Louisiana Tech University, Ruston, Louisiana, United States of America
2. Department of Mathematics and Statistics, Louisiana Tech University, Ruston, Louisiana, United States of America

*****Corresponding author

E-mail: tmurray@latech.edu (TAM)

Supplemental Information

MATSAP is user-friendly and flexible enough to meet different research requirements with respect to adjusting speed and eccentricity parameters to achieve greater sensitivity or specificity. MATSAP Threshold Optimizer can assist users in finding the optimal speed and eccentricity value thresholds based on a sample set of scored videos. These threshold values can be selected based on the nature of the data along with the sensitivity, specificity, accuracy, MCC, F-score, and area under the curve (AUC). For example if an experimenter needs to analyze SAP in 120 videos, ten of the videos can be scored by observers. Then MATSAP Threshold Optimizer can be used to find optimal speed and threshold measurements based on the scored data. Using these calculated thresholds, the 120 videos can be scored.

Optimizing Threshold in Open Field

Using the MATSAP Threshold Optimizer (Supplementary Software 1), different eccentricity and speed thresholds were explored to optimize SAP detection in the open field. Supplementary Table S1 provides a summary of this analysis. The maximum MCC of 0.6803 occurred when the speed and eccentricity values of 16 cm/s and 0.92 are chosen, respectively (Supplementary Fig. S4). The F-score was also at the maximum of 0.7164 (Supplementary Fig. S5) and the accuracy was 93.03%. At these thresholds, the sensitivity was 78.34% and the specificity was 94.89%.

Since the negative class (SAP not present) was greater than the positive class (SAP present), the MCC score favored a higher specificity. Since the positive and negative class may be more balanced in other experiments, a relatively balanced sensitivity and specificity was desired in conjunction with a high MCC. This is the rationale we used for selecting 12 cm/s and 0.90 as thresholds values for speed and eccentricity, respectively. The maximum accuracy of 93.50% occurred when the speed and eccentricity values of 12 cm/s and 0.92 were chosen, respectively (Supplementary Fig. S3). The sensitivity and specificity at these thresholds were 65.28% and 97.07%, respectively. The specificity was favored for accuracy as there were more negative classes present in this experiment. In our application, we do not desire high specificity at the cost of losing sensitivity. The maximum AUC of 0.9077 occurred when the speed and eccentricity values of 19 cm/s and 0.91 are chosen, respectively (Supplementary Fig. S6). At these thresholds, the sensitivity was 92.58% and the specificity was 88.92%. A specificity slightly higher than sensitivity would be preferable since in practice it is more likely that SAP will *not* be present than present.

Optimizing Threshold in Elevated Plus Maze

MATSAP Threshold Optimizer was also used to explore different eccentricity and speed thresholds in the elevated plus maze. Supplementary Table S2 provides a summary of this analysis. The maximum MCC of 0.7016 occurs when the speed and eccentricity values of 8 cm/s

and 0.89 are chosen, respectively (Supplementary Fig. S8). At these thresholds, the sensitivity was 85.96% and the specificity was 85.30. The accuracy, F-score, and AUC were 85.56%, 0.8194, and 08563, respectively. The maximum accuracy of 85.63% occurred when the speed and eccentricity values of 8 cm/s and 0.90 were chosen, respectively (Supplementary Fig. S7). The sensitivity and specificity at these thresholds were 79.41% and 89.46%, respectively. The maximum F-score and maximum AUC both occur when the speed threshold was 9 cm/s and the eccentricity threshold value was 0.89 (Supplementary Fig. S9 and S10). The sensitivity and specificity values were 87.03% and 84.38%, respectively.

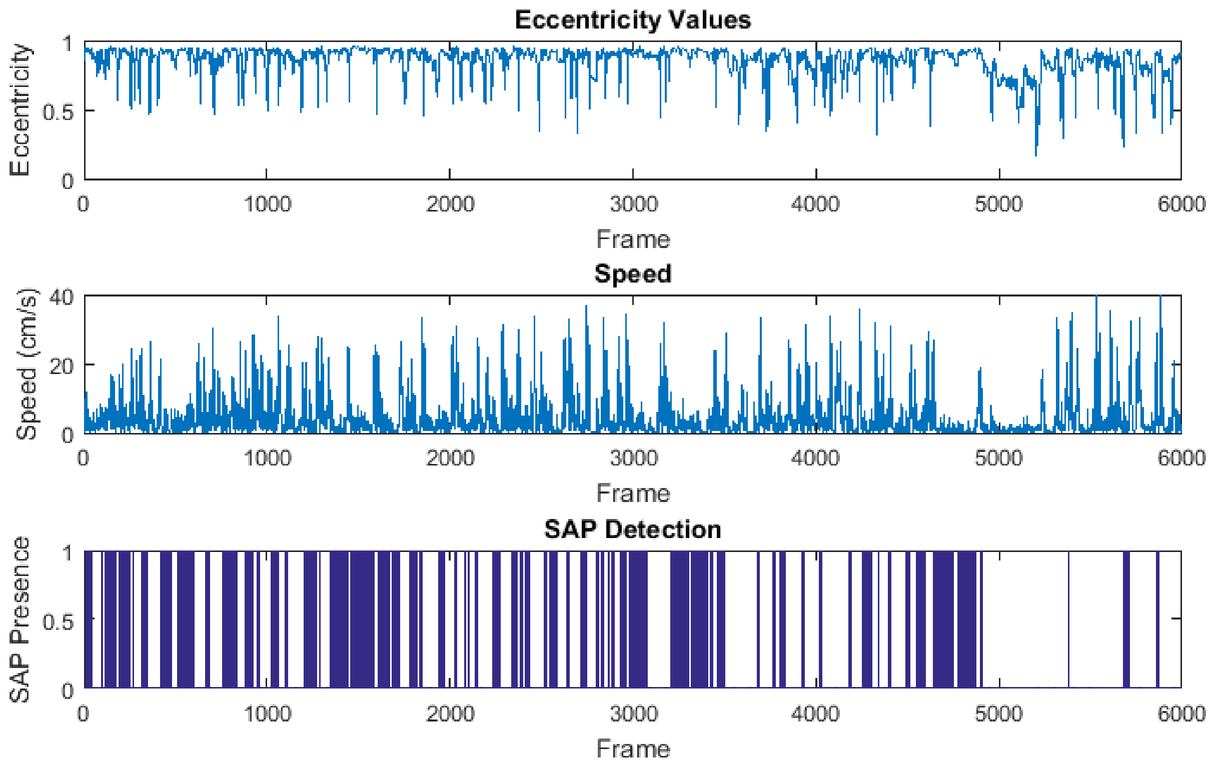

Supplementary Figure S1

Output plots for elevated plus maze video.

SAP behavior is more uniform throughout time in the EPM in comparison to the OF.

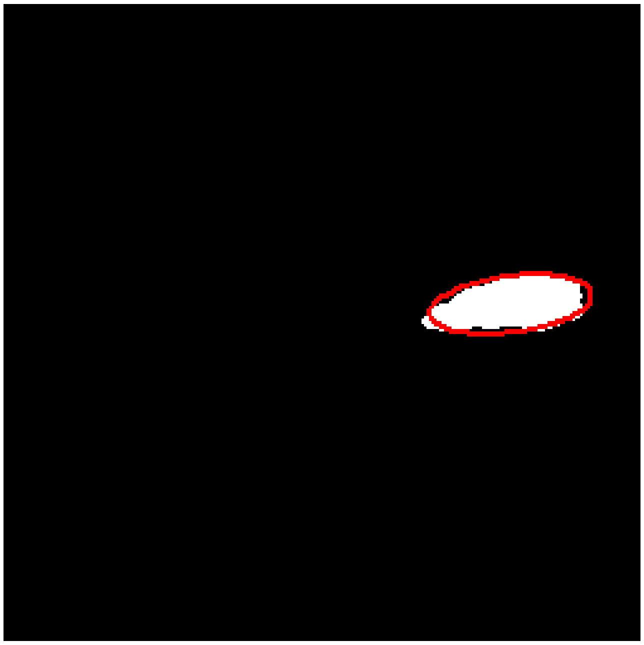

Supplementary Figure S2

Output of an open field video (single frame).

This image demonstrates that the image analysis successfully formed an ellipse around the body of the rodent.

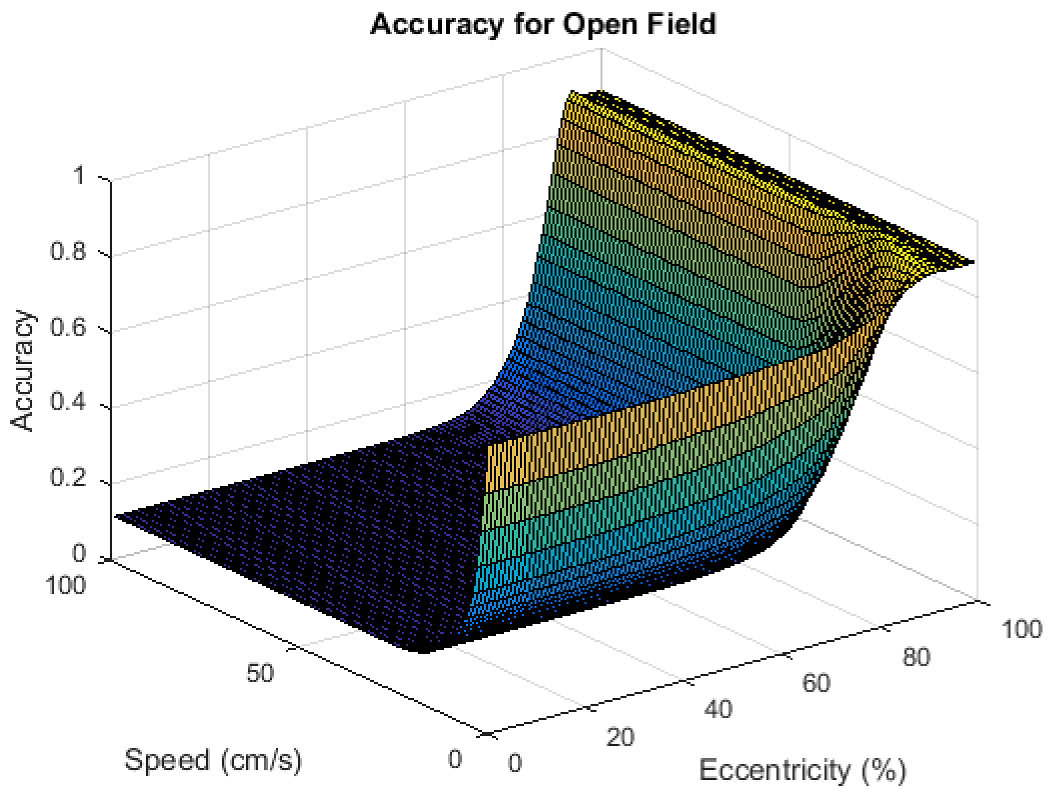

Supplementary Figure S3

Accuracy of MATSAP analyzing open field videos.

The maximum accuracy of 93.03% occurred with a speed threshold of 12 cm/s and an eccentricity threshold of 92%.

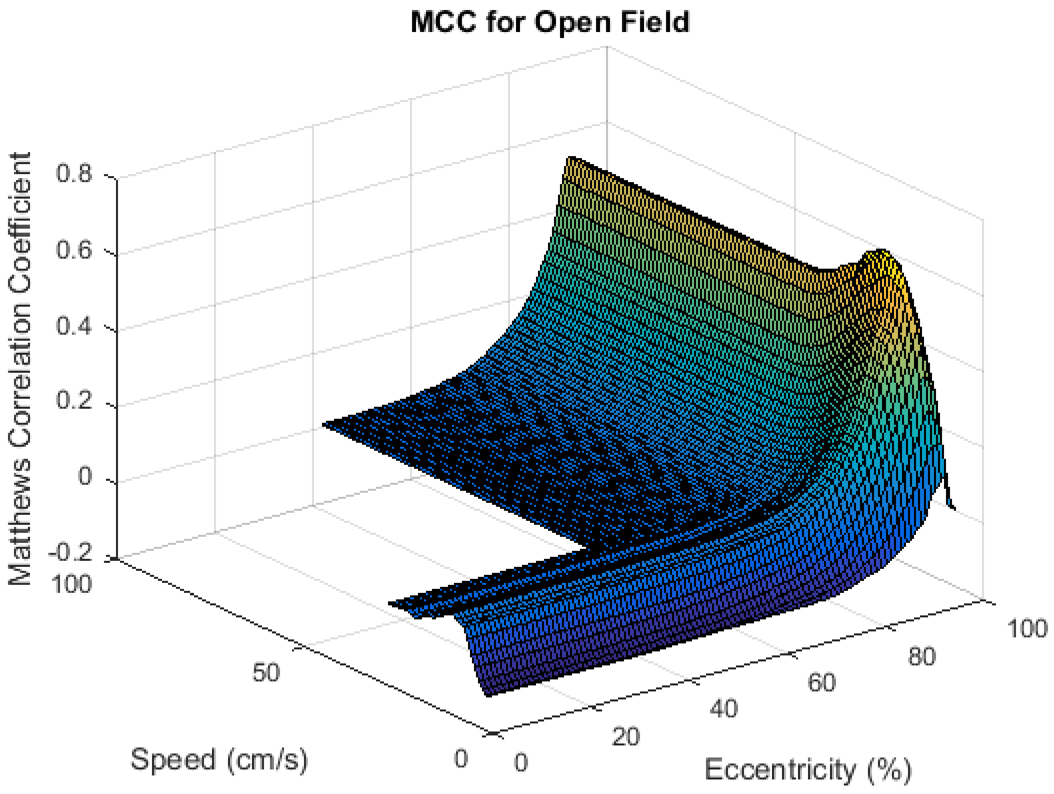

Supplementary Figure S4

MCC of MATSAP analyzing open field videos.

Matthews correlation coefficient (MCC) values when analyzing open field videos at different speed and eccentricity thresholds. The maximum MCC of 0.68 occurred with a speed threshold of 16 cm/s and an eccentricity threshold of 92%.

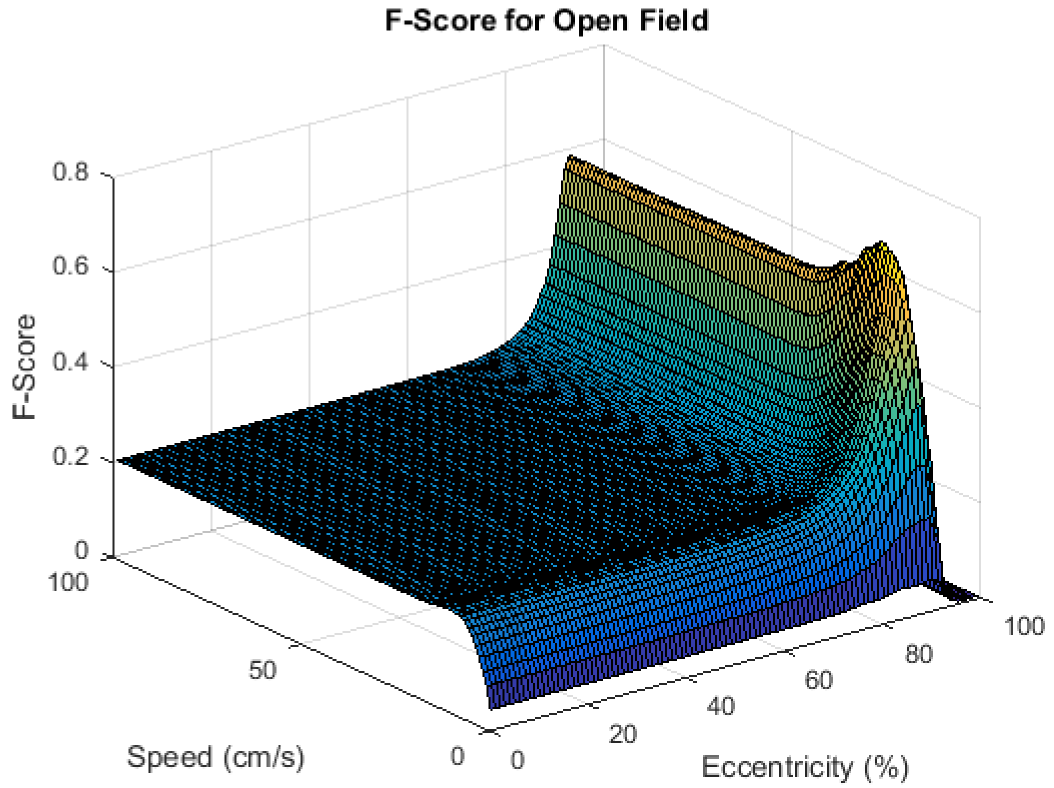

Supplementary Figure S5

F-score of MATSAP analyzing open field videos.

The maximum F-score of occurred with a speed threshold of 16 cm/s and an eccentricity threshold of 92%.

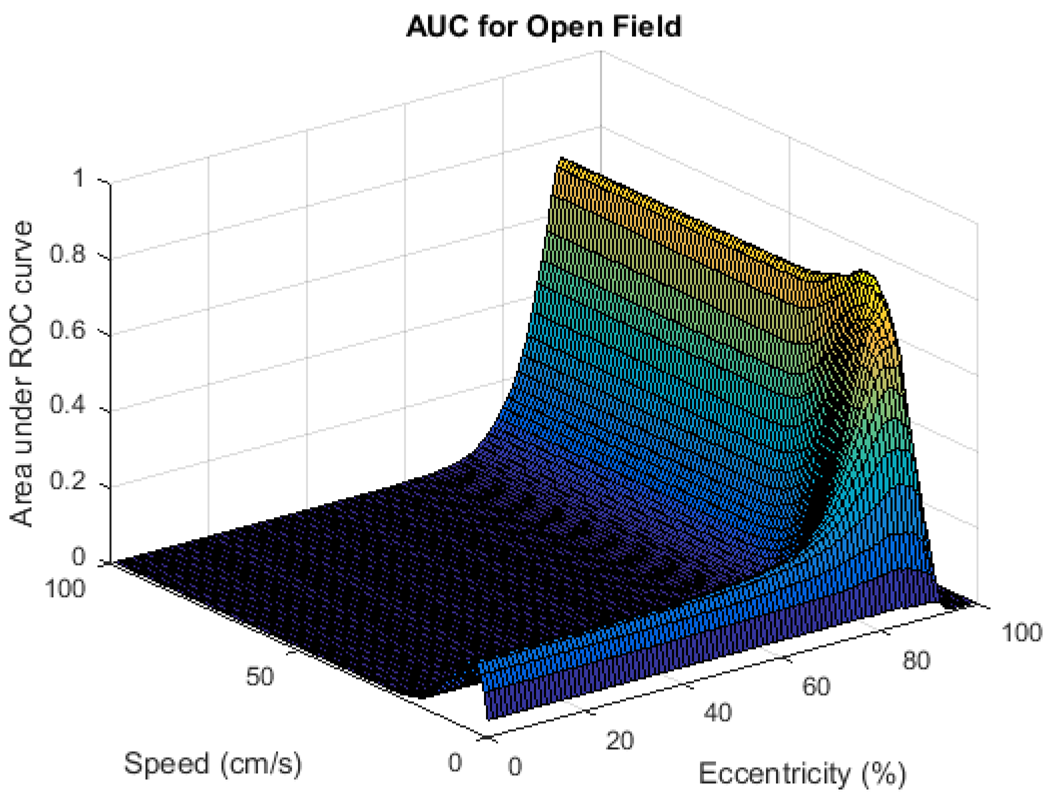

Supplementary Figure S6

Area under the ROC curve for MATSAP analyzing open field videos.

The maximum AUC of 0.9077 occurred with a speed threshold of 19 cm/s and an eccentricity threshold of 91%.

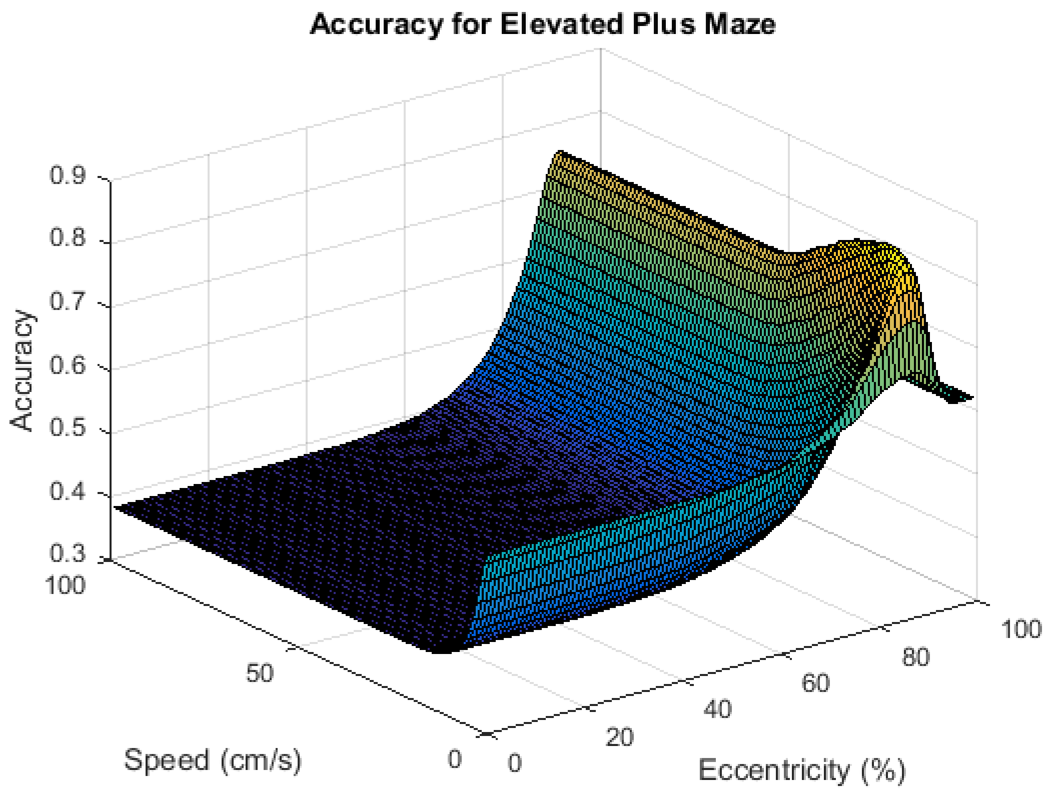

Supplementary Figure S7

Accuracy of MATSAP analyzing elevated plus maze videos

The maximum accuracy of 85.63% occurred with a speed threshold of 8 cm/s and an eccentricity threshold of 90%.

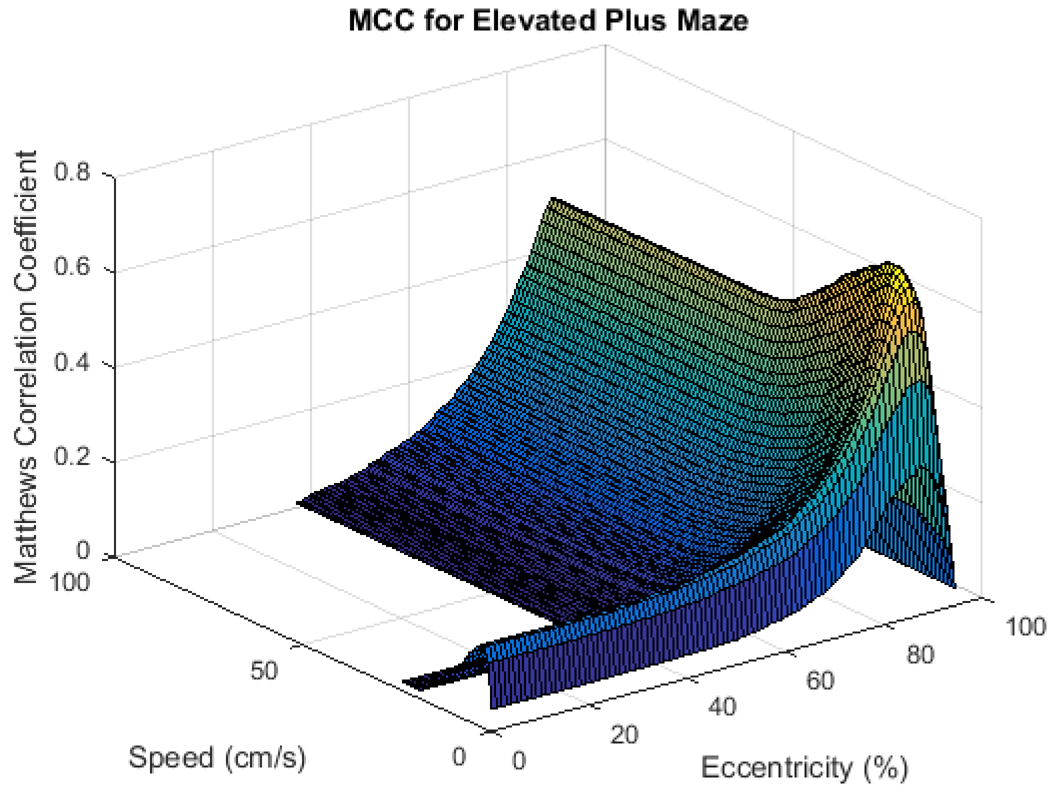

Supplementary Figure S8

MCC of MATSAP analyzing elevated plus maze videos.

Matthews correlation coefficient values when analyzing elevated plus maze videos at different speed and eccentricity thresholds. The maximum MCC of 0.70 occurred with a speed threshold of 8 cm/s and an eccentricity threshold of 89%.

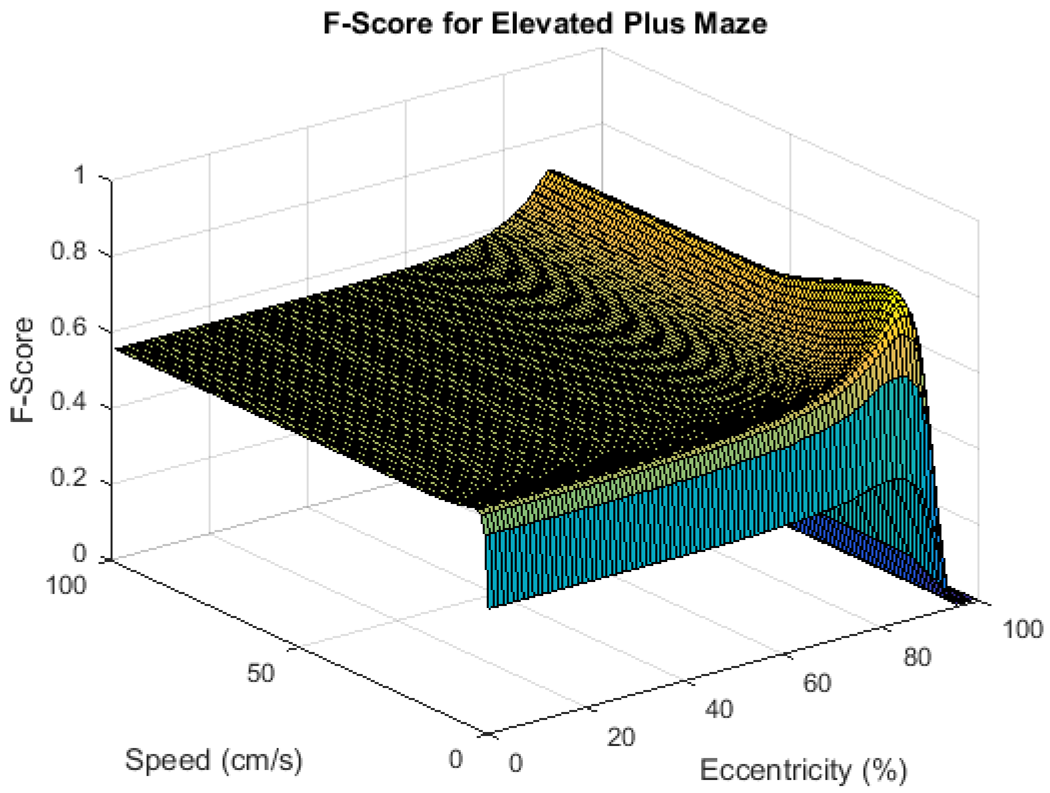

Supplementary Figure S9

F-score of MATSAP analyzing elevated plus maze videos.

The maximum F-score of occurred with a speed threshold of 16 cm/s and an eccentricity threshold of 92%.

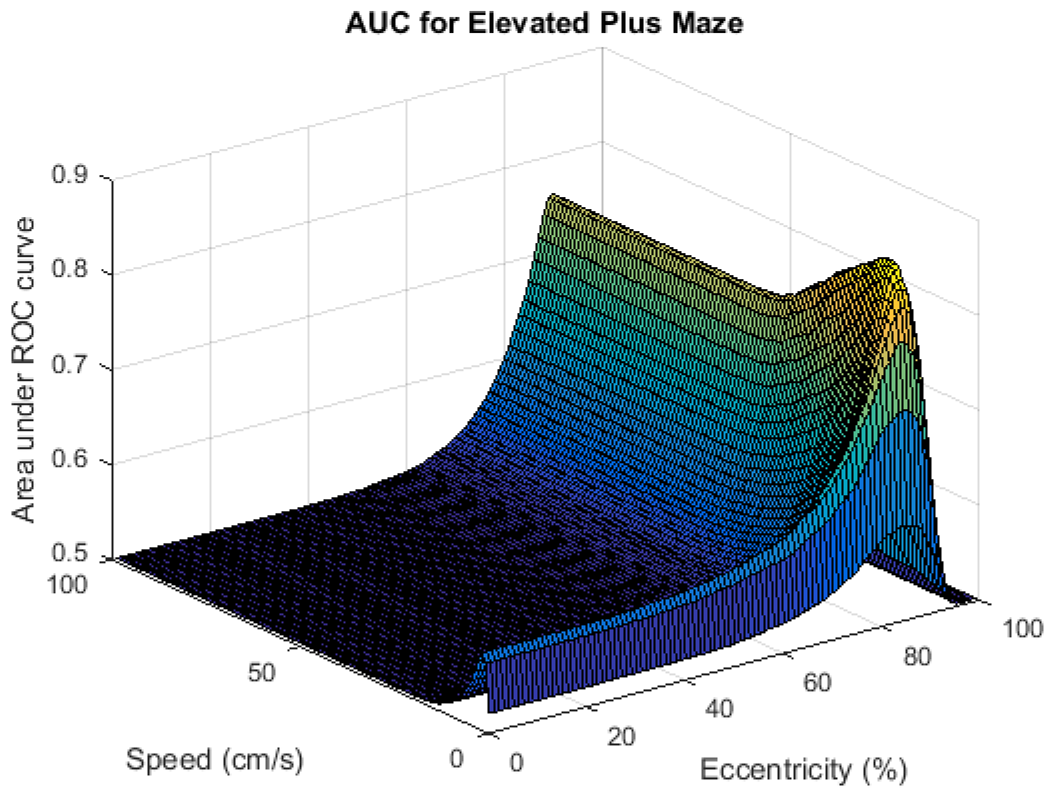

Supplementary Figure S10

Area under the ROC curve for MATSAP analyzing open field videos.

The maximum AUC of 0.7343 occurred with a speed threshold of 9 cm/s and an eccentricity threshold of 89%.

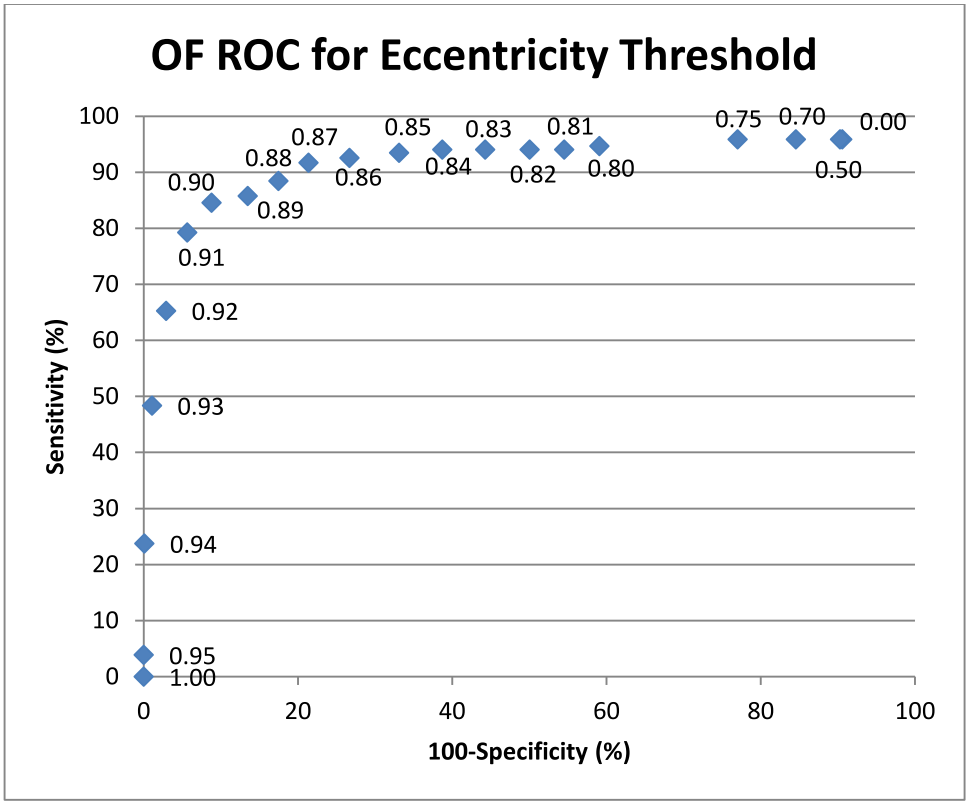

Supplementary Figure S11

ROC curve for eccentricity threshold when speed threshold is set at 12 cm/s for open field.

The chosen eccentricity threshold value of 0.90 can be found on the top left of the ROC curve indicating a reasonable value.

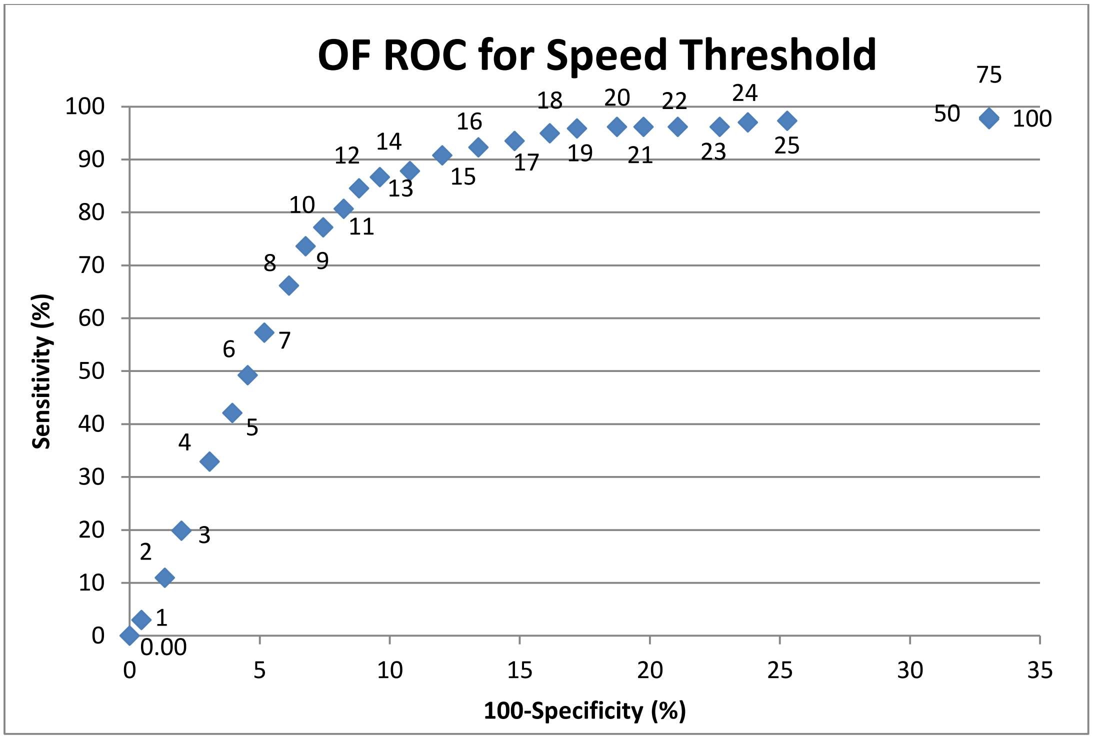

Supplementary Figure S12

ROC curve for speed threshold when eccentricity threshold is set at 0.90 for open field.

The chosen speed threshold value of 12 cm/s can be found on the top left of the ROC curve indicating a reasonable value.

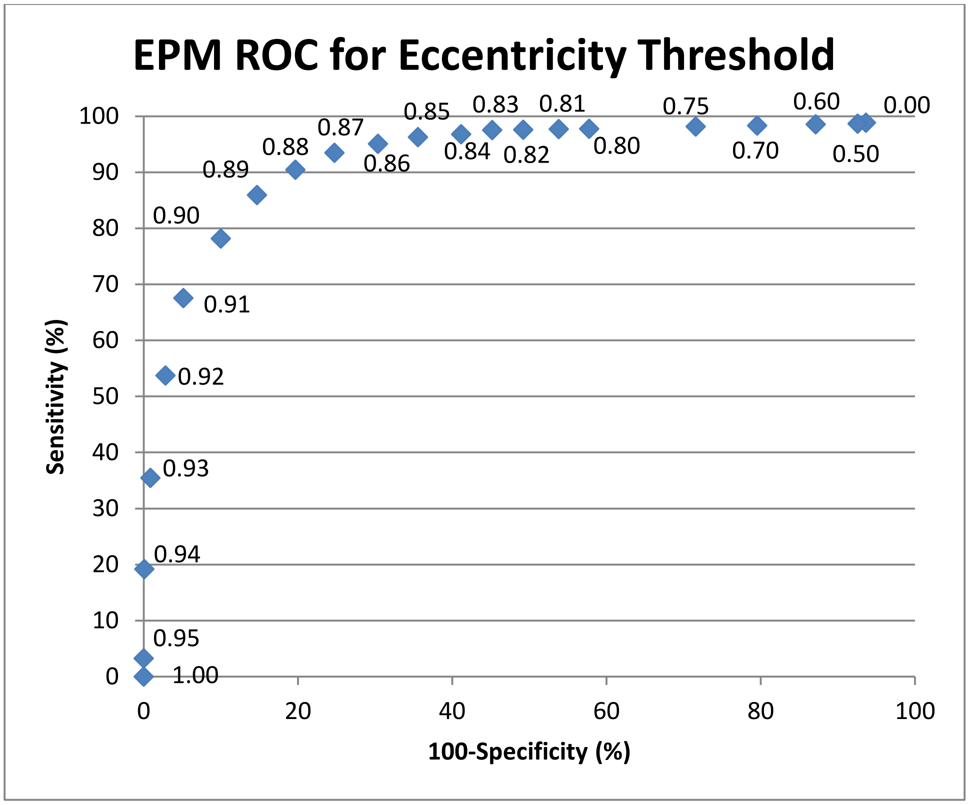

Supplementary Figure S13

ROC curve for eccentricity threshold when speed threshold is set at 8 cm/s for elevated plus maze.

The chosen eccentricity threshold value of 0.89 can be found on the top left of the ROC curve indicating a reasonable value.

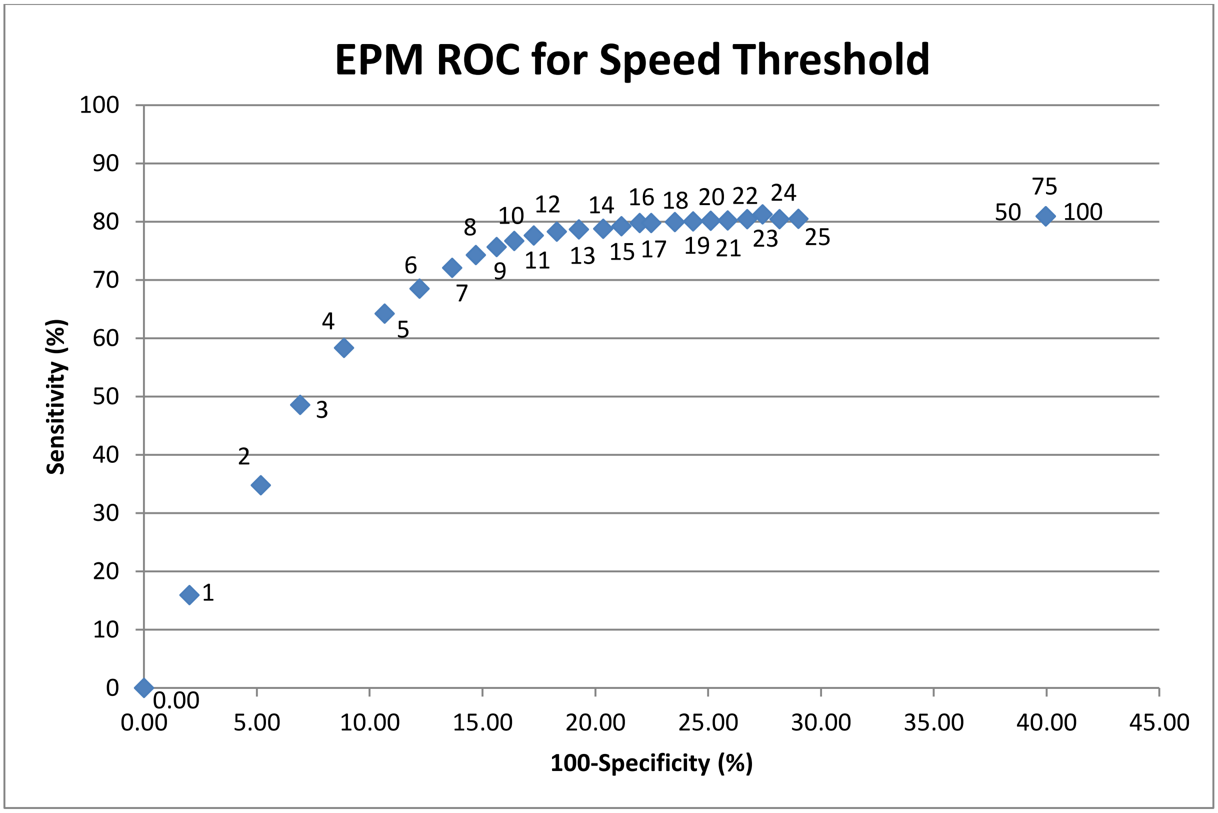

Supplementary Figure S14

ROC curve for speed threshold when eccentricity threshold is set at 0.89 for elevated plus maze.

The chosen speed threshold value of 8 cm/s can be found near the top left of the ROC curve indicating an acceptable value.

SUPPLEMENTARY TABLES

Supplementary Table 1

Threshold optimization for open field.

Statistical measurements for MATSAP analyzing Open Field								
	Speed (cm/s)	Eccentricity (%)	Sensitivity (%)	Specificity (%)	Accuracy (%)	MCC	F-score	AUC
max MCC	16	92	78.34	94.89	93.03	0.6803	0.7164	0.8662
max Accuracy	12	92	65.28	97.07	93.50	0.6583	0.6929	0.8118
max F-score	16	92	78.34	94.89	93.03	0.6803	0.7164	0.8662
max AUC	19	91	93.47	88.06	88.67	0.6310	0.6495	0.9077
chosen	12	90	84.87	91.18	90.47	0.6339	0.6667	0.8802

Supplementary Table 2

Threshold optimization for elevated plus maze.

Statistical measurements for MATSAP analyzing Elevated Plus Maze								
	Speed (cm/s)	Eccentricity (%)	Sensitivity (%)	Specificity (%)	Accuracy (%)	MCC	F-score	AUC
max MCC	8	89	85.96	85.30	85.56	0.7016	0.8194	0.8563
max Accuracy	8	90	79.41	89.46	85.63	0.6897	0.8102	0.8407
max F-score	9	89	87.03	84.38	85.39	0.7009	0.8196	0.8570
max AUC	9	89	87.03	84.38	85.39	0.7009	0.8196	0.8570
chosen	8	89	85.96	85.30	85.56	0.7016	0.8194	0.8563

MATSAP Threshold Optimization Guide

This guide will assist users in optimizing the eccentricity and speed threshold values for a particular experiment.

1. Manual Scoring: Evaluate the presence of SAP in sample videos

    a. Classify the presence of SAP in videos on a second by second basis

        i. When SAP is present, give the classification is “1”. When SAP is not present, give the classification “0”.

        ii. Record classifications for each second of video. List the scores (0 or 1) in consecutive order by time.

        iii. Compile consensus classifications for all sample videos into a single spreadsheet column. Place the classifications in order of the sample videos arranged in alphabetical/numerical order.

    b. Use 2 expert raters (inter-rater reliability >0.90) or 4 – 5 trained, but non-expert raters (inter-rater reliability >0.75) to assess sample videos.

    c. Calculate the consensus classification at each second via majority voting between raters in step b.

    d. In our study, we evaluated ten 5 minute open field videos at 10 fps. We had 5 observers classify if SAP was present at each second, totaling 3000 seconds.

    e. Save the single column spreadsheet containing the binary classification of SAP for all sample videos as a .csv file named “Consensus.csv”.

2. Video Preparation

    a. Convert videos into multi-TIFF files

        i. Convert video files into AVI files (ImageJ can read AVI videos) and adjust fps if needed. This can be done with Any Video Converter, AVS Video Converter, or other video converting program.

        ii. Use ImageJ to convert files into multi-TIFF. If needed, crop image to known dimensions and create a background image (see Appendix for detailed instructions).

    b. Place multi-TIFF files that will be used to optimize the threshold into a folder without any other TIFF files present.

3. Run MATSAP Threshold Optimizer

a. Follow instructions as prompted

b. If the program is running slowly or you receive a memory error, consider reducing the sample size. Try using multi-TIFF files with lower fps. Alternatively, try using fewer or shorter sample videos (this would require adjusting the consensus data, so lowering the fps may be preferable).

c. Once the videos are analyzed, you should obtain 8 figures and a summary table similar to what is depicted below.

	Speed	Eccentricity	Sensitivity	Specificity	Accuracy	MCC	Fscore	AUC
max MCC	16	92	78.3	94.9	93	0.68	0.72	0.87
max Accuracy	12	92	65.3	97.1	93.5	0.66	0.69	0.81
max F-score	16	92	78.3	94.9	93	0.68	0.72	0.87
max AUC	19	91	93.5	88.1	88.7	0.63	0.65	0.91

d. Enter the commands below in the command window of MATLAB to explore additional speed and eccentricity threshold values that are not provided by the table.

Commands (case-sensitive):

MCC(S,E)
Accuracy(S,E)
Sensitivity(S,E)
Specificity(S,E)
Fscore(S,E)
AUC(S,E)

When entering the above commands in the MATLAB command window, enter numerical values for S and E (S= Speed threshold value (cm/s), E= Eccentricity Threshold value (%)).

Example:

Let’s say we want to investigate the MCC when the eccentricity threshold is 90% and the speed threshold is 12 cm/s. The command below would be written in the MATLAB command window.

MCC(12,90)

The MCC value will then be given and the MATLAB command window would be ready to receive more commands from the user.

Appendix

1. File>Open (Ctrl + O)
    i. Select AVI video you wish to convert to multi-TIFF
    ii. Select “OK” when “AVI Reader” dialog prompt appears.
2. If you need to crop image,
    i. Select the region you wish to crop
    ii. Image>Crop (Ctrl + Shift + X)
3. Create a background frame,
    i. Move to the last frame of video (scroll slide bar to the right)
    ii. Image>Stacks>Add Slice
    iii. Move to the previous frame
    iv. Edit>Selection>Select All (Ctrl+A)
    v. Edit>Copy (Ctrl + C)
    vi. Move to the last frame
    vii. Edit>Paste (Ctrl + V)
    viii. Take note of the location of the rodent and move to a previous frame where rodent is not present in that particular location.
    ix. Select the empty region where rodent was present in the last frame.
    x. Edit>Copy (Ctrl + C)
    xi. Move to the last frame without deselecting region
    xii. Edit>Paste (Ctrl + V)
